# Quantitative Approach to Explore Regulatory T Cell Activity in Immuno-Oncology

**DOI:** 10.3390/pharmaceutics16111461

**Published:** 2024-11-15

**Authors:** Alejandro Serrano, Sara Zalba, Juan Jose Lasarte, Iñaki F. Troconiz, Natalia Riva, Maria J. Garrido

**Affiliations:** 1 Department of Pharmaceutical Sciences, School of Pharmacy and Nutrition, University of Navarra, 31008 Pamplona, Spain; aserranoa@unav.es (A.S.); szalbaot@unav.es (S.Z.); itroconiz@unav.es (I.F.T.); 2 Navarra Institute for Health Research (IdisNA), 31008 Pamplona, Spain; 3 Program of Immunology and Immunotherapy, Center for Applied Medical Research (CIMA), University of Navarra, 31008 Pamplona, Spain; jjlasarte@unav.es; 4 Institute of Data Sciences and Artificial Intelligence (DATAI), University of Navarra, 31008 Pamplona, Spain

**Keywords:** Treg cells, QSP model, immunotherapy, tumor growth dynamics, computational immune framework

## Abstract

The failure of immunotherapies in cancer patients is being widely studied due to the complexities present in the tumor microenvironment (TME), where regulatory T cells (Treg) appear to actively participate in providing an immune escape mechanism for tumors. Therefore, therapies to specifically inhibit tumor-infiltrating Treg represent a challenge, because Treg are distributed throughout the body and provide physiological immune homeostasis to prevent autoimmune diseases. Characterization of immunological and functional profiles could help to identify the mechanisms that need to be inhibited or activated to ensure Treg modulation in the tumor. To address this, quantitative in silico approaches based on mechanistic mathematical models integrating multi-scale information from immune and tumor cells and the effect of different therapies have allowed the building of computational frameworks to simulate different hypotheses, some of which have subsequently been experimentally validated. Therefore, this review presents a list of diverse computational mathematical models that examine the role of Treg as a crucial immune resistance mechanism contributing to the failure of immunotherapy. In addition, this review highlights the relevance of certain molecules expressed in Treg that are associated with the TME immunosuppression, which could be incorporated into the mathematical model for a better understanding of the contribution of Treg modulation. Finally, different preclinical and clinical combinations of molecules are also included to show the trend of new therapies targeting Treg.

## 1. Introduction

Immunotherapy in oncology enhances the ability of the immune system to control or eliminate cancer cells, thus changing the paradigm of cancer treatment [1]. However, tumor cells can develop various resistance or immune evasion mechanisms, such as the introduction of mutations and/or upregulation of immune checkpoints, in particular programmed death-ligand 1 (PD-L1). This upregulation contributes to generating an immunosuppressive tumor microenvironment (TME) that enables tumor growth and proliferation [2,3,4,5].

**Figure 1:**
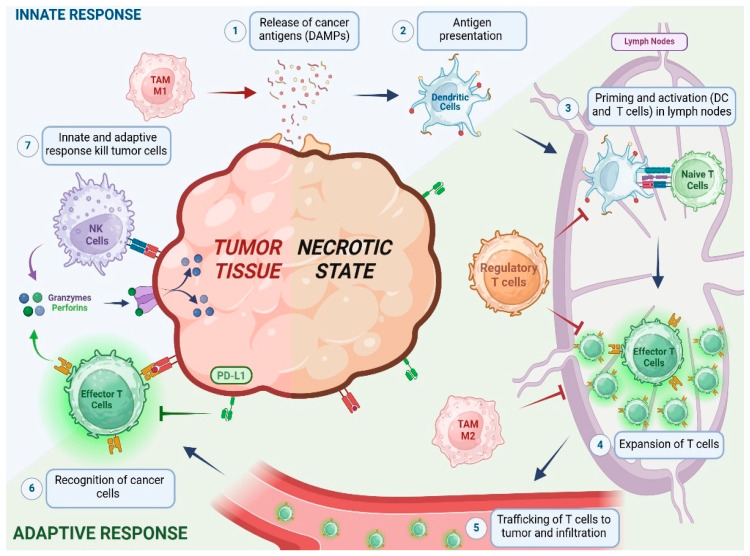
Scheme of cancer cycle immunity. Tumor antigens derived from cell death are engulfed by DCs and presented to naive T cells, resulting in the activation of effector cytotoxic T cells (CD8^+^) capable of inducing tumor cell apoptosis and generating a humoral response. This process promotes the secretion of pro-inflammatory cytokines and DAMPs that enhance T cell activation.

The relationship between tumor growth dynamics and changes in the TME is highly complex due to the behavior of the immune system, which is mainly responsible for this complexity. Figure 1 illustrates this relationship in a simplified way, which is based on the cancer-immunity cycle described by Mellman et al. [6]. Briefly, dendritic cells (DCs) recognize and engulf neoantigens and damaged associated molecular patterns (DAMPs) produced by dead tumor cells, activating the circulating naive T cells that link the innate and adaptive immune responses. However, it is the balance between immune activation and suppression that determines whether a tumor will proliferate or be eliminated. The antitumor response is induced by DC, M1 macrophages, CD4^+^ T cells, and cytotoxic CD8^+^ T cells [7,8,9,10]. On the other hand, tumor resistance is derived from M2 macrophages, myeloid-derived suppressor cells (MDSCs), and regulatory T cells (Treg) [11,12,13].

This simplified framework on the ICI is intended to illustrate the key events that trigger the antitumor immune effect. It describes the integration of many cell populations that can be modulated by the action of cytokines, chemokines and growth factors that play a relevant role by interacting with these cells and changing the immunophenotype of the tumor. Indeed, the dynamics of immune cells in the TME is strongly influenced by the plasticity of some of them, particularly Treg and myeloid cells infiltrating the tumors. This, together with the complexity of the immune synapse between different cell subpopulations, conforms to a complex scenario [14].

In this context, the design of strategies to improve antitumor efficacy requires substantial knowledge of these complex interactions between the immune system and cancer cells, and the identification of the essential mechanisms that need to be inhibited or stimulated to achieve total tumor rejection and generate immune memory. Given these unmet medical requirements in immune oncology, mathematical models stand out for their ability to describe and quantify the dynamics of different physio-pathological processes involved in tumor progression and the activity of immunotherapy.

Different types of models have been reported in the literature, including empirical and semi-mechanistic models which aim to describe and/or predict tumor growth. These models have provided a good description of tumor growth kinetics after different treatments in animals and patients. They show interesting applications for the development of new agents and propose different drug combinations or even new dose regimens to reduce the toxicities induced by chemotherapy or radiotherapy. However, in order to integrate the knowledge of the mechanisms triggered by tumors, therapies, and the immune system, the model structure requires greater complexity, which is addressed by the quantitative system pharmacology (QSP) approach.

The QSP approach in immune oncology (IO) represents a differentiated scientific and bioinformatic tool that incorporates physiology, physiopathology, inter-individual variability, and therapeutic activity to describe and characterize the interactions between cancer cells and immune cells, in a granular way. Importantly, QSP model building is, generally, based on multi-scale information integrating relevant biomarkers and cell populations, although these models require certain simplifications of the events generated in the TME, which nevertheless reflect the main mechanisms that trigger the immune response.

In some of these models, the activity of immunotherapy has been adapted by incorporating the effect of activated tumor-specific cytotoxic CD8^+^ T cells responsible for tumor elimination together with the immune resistance response associated with the failure of many immunotherapeutic approaches, mainly due to the presence of tumor-infiltrating Treg. Although serious efforts have been made to identify up- or down-regulated biomarkers in Treg, this information has not yet been elucidated and integrated in a quantitative framework [15].

Therefore, this review summarizes a list of quantitative computational models that relate Treg activity to antitumor immunity, exploring the impact of some relevant mechanisms involved in IO therapies. Furthermore, different therapeutic strategies to counteract the immunoresistance effect of Treg are briefly discussed as a possible contribution to the development of QSP models.

## 2. Regulatory T Cells in TME

Treg maintain physiological immune homeostasis by balancing excessive immune responses with suppression of autoimmune responses.

Treg belong to the CD4^+^ T cell population and are characterized by the expression of CD4 and CD25 (or IL-2 receptor) on their surface, and the intracellular nuclear transcription factor Forkhead box P3 (Foxp3), which controls Treg proliferation and activity. However, both biomarkers, Foxp3 and CD25, are also transiently expressed on activated effector T cells (cytotoxic CD8^+^ T cells). Therefore, it is relevant to selectively identify Treg to study their role in IO. In this sense, Cytotoxic T-Lymphocyte-Associated Protein 4 (CTLA-4) is one of the preferentially overexpressed biomarkers on the surface of Treg, which has led to the synthesis of a specific monoclonal antibody against Treg (Ipililimab). However, other molecules are under investigation because of their relationship with their immunosuppressive activity, which can contribute to the development of novel inhibitors of Treg. Thus, Treg can be classified based on their biomarker expression and cytokine release patterns. A summary of such biomarkers is provided in Supplementary Table S1.

It is known that Treg can migrate to specific tissues in response to chemokine signaling and differentiate into CD25^high^Foxp3^high^, a characteristic of immunosuppressive Treg (iTreg), which represents one of the immune escape mechanisms developed by the tumors [16,17].

Chemokines such as CCL17 and CCL22 are endogenous ligands that selectively bind to the CCR4 receptor expressed on activated Treg, promoting Th2 response and thereby facilitating Treg tumor infiltration [18,19]. Similarly, CCL18 or CCL1 bind to the CCR8 receptor, which is strongly expressed on tumor-infiltrating Treg, and promote Treg proliferation and activation through Foxp3 upregulation [20]. Therefore, the chemokine receptors, CCR4 and CCR8, are particularly involved in Treg modulation and immunotherapy efficacy, as these Treg release immunosuppressive cytokines such as IL-10, TGFβ, and IL-35 within the TME, competing with the effector CD8^+^ T cells for IL-2 by upregulating the expression of CD25. This activity promotes the depletion of NK cells and disrupts the activation and proliferation of effector CD8^+^ T cells, which become exhausted, inducing an immunotolerant and immunosuppressive TME. As a consequence of this process, iTreg infiltration in the TME is associated with a poor prognosis and the failure of immunotherapies [21].

Since efficacy of immunotherapies lies in the enhancement of immune surveillance, appropriate modulation of functional Treg activity may improve this mechanism and thus clinical response. To address this, mathematical modeling provides a useful tool to better explore these complexities using an in silico approach to identify mechanisms that could explain the observed clinical or preclinical results [22].

## 3. Relevance of Quantitative Mathematical Models to Explore Treg Activity in Tumor Growth

Interest in characterizing tumor growth using quantitative approaches, including empirical, semi-mechanistic and QSP models, has grown over the years. One of the pivotal mathematical models is the data-driven pharmacokinetic/pharmacodynamic (PKPD) model, which is capable of describing tumor growth and identifying significant covariates affecting some model parameters under different scenarios through in silico simulations [23]. The main goal of such a PKPD model is the screening of tumor sensitivity to drugs and drug distribution in the tumor, relying on data from immunosuppressed animal models (i.e., xenograft mouse), and thus not considering immunological aspects.

Notably, the inclusion of immunoresistance mechanisms and the antitumor effects exerted by effector CD8^+^ T cells were later accounted for by using data from syngenic murine tumor models. This animal model allows for the identification of inter-subject variability in the immune response and the stratification of individuals into responders and non-responders [24]. In addition, these models have provided a framework for testing the effects of different therapeutic agents including vaccines, Toll-like receptor (TLR) agonists, and immune checkpoint inhibitors, among others. However, the role of pro-tumor factors such as infiltrating Treg, MDSCs, and PD-L1 upregulation were implicitly modeled due to the lack of specific information on lymphocyte populations and biomarker levels. This highlights the critical need for experimental data to elucidate the underlying pro-tumor mechanisms.

In recent years, new experimental methodologies using functional and phenotypic assays have greatly improved the ability (1) to study the temporal characteristics of tumor and immune cell progression, (2) to capture the heterogeneity of tumors and TME [24], and (3) to provide more comprehensive in vitro and in vivo information on novel therapies. These advances challenge the incorporation of the data obtained into mechanistic computational models, which occurs in the QSP model structure.

**Figure 2:**
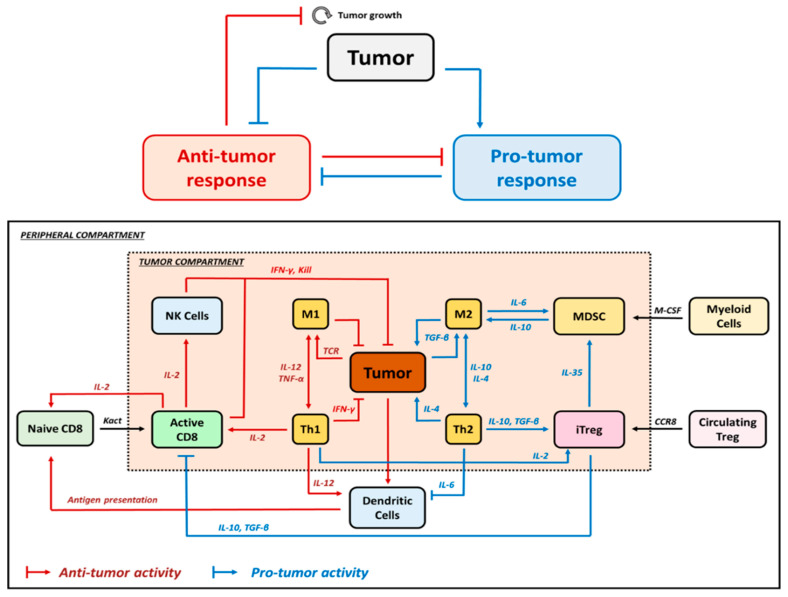
Processes involved in tumor elimination (represented in red) or tumor progression (represented in blue). Stimulatory mechanisms to induce antitumor effect must overcome inhibitory activities. Intra-tumoral infiltration of activated CD8^+^ T cells triggers tumor cell death through the action of interferon-gamma (IFN-γ), and granzyme B. However, the tumor evades this control by inducing specific immunoresistance mechanisms, particularly the proliferation of Treg cells and the upregulation of PD-L1 on the surface of tumor cells, leading to effector cell exhaustion.

Understanding the TME, its elements, and the immune framework is essential to determine the impact of effector CD8^+^ T cells alongside Treg. Figure 2 depicts the main processes involved in either (i) tumor elimination (represented in red) where CD8^+^ T cells are essential, or (ii) tumor progression (represented in blue) where Treg are the main responsible elements. In addition, coordination across lymphocytes CD8^+^, CD4^+^, Treg, NK cells, macrophages M1 and M2, MDSCs and DCs is required to regulate the development and progression of the disease. Integrating these processes with their corresponding biomarkers into a mathematical framework that replicates the most complex immunological state triggered by the presence of a tumor is the most challenging exercise in predicting the final immune response.

Several promising biological markers have been identified to determine the balance between antitumor immune response and resistance mechanisms. In particular, an increased intra-tumoral CD8^+^/Treg ratio has been identified as a predictor of tumor shrinkage in preclinical studies and has also been translated into clinical trials [25]. An increase in activated CD8^+^ T cells shifts the balance in favor of the immune system to achieve an antitumor response. However, the presence of Treg and their balance with CD8^+^ T cells have not fully explained clinical outcomes. This has led to a great interest in preclinical studies to investigate the role of Treg inhibition, stimulation, or depletion within the TME, which ultimately determines the efficacy of immunotherapeutic approaches in cancer [26]. It should be noted that cytokines and chemokines are also relevant players in Treg modulation, and therefore, their integration into these computational QSP models is necessary. On the other hand, the role of other immune cells present in the TME, such as the T helper cells and macrophage subsets that control the Th1/Th2 and M1/M2 ratios, respectively, in tumor clearance is not yet fully understood [27].

Different QSP quantitative frameworks have been published in the literature partially covering the pro- and antitumor response elements described in Figure 2 [28]. In order to explore and consider the role of Treg in cancer in a comprehensive manner, we focused our review on those that included the antitumoral effect of Treg in immunotherapy and applied the following inclusion criteria: (1) preclinical and clinical studies, (2) time evolution dynamics of cell populations (described by ordinary differential equations, ODEs), and (3) tumor dynamic profiles as the main outcome. We also included articles studying longitudinal clinical data on cytokines and relevant lymphocyte populations. A total of six articles combining preclinical and clinical data were selected, numbered and presented in Table 1, according to increasing complexity in terms of mechanistic aspects, cell populations, assay complexity, spatial components (tumor vasculature, tumor compartment), and tested therapies.

The first article, reported by Kronik et al., presents a mathematical non-spatial model developed to describe cellular immunotherapy for melanoma using clinical trial data integrating CD8^+^ T cells and cytokines [29]. The model includes melanoma cells that express immunogenic antigens in the context of the major histocompatibility complex I (MHC I) molecules, but also secrete the pro-tumor factor TGF-β, which inhibits T cell activity, implicitly representing the effects of Treg. The tested treatment consists of adoptive T cell therapy, based on ex vivo expanded tumor-specific T cell infusion that lyses their target and secretes the antitumor factor IFN-*γ*, which has a positive feedback on MHC I molecules, increasing the cytotoxic T lymphocyte (CTL)-mediated antitumor effect. In addition, treatment with IL-2 infusions prolongs the persistence of infused CTL. The combination of both therapies promotes a higher antitumor efficacy than adoptive T cell therapy alone, with assumable adverse effects. Tumor growth rate and tumor size are essential in predicting the outcome of therapy, and this mathematical model supports the decision of the most favorable schedule for each patient.

Other studies have reported models focusing on one [30] or on parallel [31] pathways of inhibitory factors induced by Treg, TGF-β and/or IL-10 that promote tumor growth and reduce the cytotoxicity of effector cells (i.e., CD8^+^ cells and others such as DC, CD4^+^ helper T cells, and IL-2). In the model reported by Wilson et al., using data from a TC1 murine tumor model, the authors considered two types of interactions between tumor cells and T cells: (1) the interaction with Treg through TGF-β release to investigate the anti-TGF-β treatment, and (2) the interaction with CD8^+^ cells, by using cancer vaccines as effector cell stimulators [30]. The authors conclude that tumor elimination requires combined immunotherapy treatments capable of working through different mechanisms since vaccine monotherapy is not sufficient to eradicate the tumor.

Increasing the complexity of the model, Robertson-Tessi et al. considered different simultaneous pathways of pro-tumor effects [31]. The model, based on in silico multi-scale data from tumors with different growth rates and antigenicity, estimates and simulates typical time profiles of tumor growth by determining the relevance of various immunosuppressive mechanisms at the different stages of tumor growth. In addition to TGF-β-induced immunosuppression, this model accounts for the conversion of CD4^+^ helper T cells into Treg, and the evaluation of dendritic cell therapy. The model structure allows for tumor escape, which is mainly supported by the immunosuppression conferred by TGF-β (greater effects with larger tumor sizes), the presence of Treg at all tumor growth stages, and the limited access of immune cells to the TME at large tumor sizes (sufficient tumor vascularization). Interestingly, for a given tumor growth rate, there is a specific antigenicity and optimal dose of transfused dendritic cells that lead to an adequate response of the immune system that is large enough to affect the tumor, but small enough to avoid excessive Treg promotion and suppressive effects.

In addition to different cytokines and the most representative immune cell populations involved in immunotherapy, the role of M1 and M2 macrophages in tumor growth, and the use of the M2/M1 ratio as a pro-tumor biomarker, has garnered considerable attention in recent years. Den Breems et al. reported a mathematical model that analyzes the stimulation of immunity by Th1 and M1 cells, and their complementary immunosuppressive pro-tumor response derived from M2 and Th2 cells, to describe data from a murine melanoma model [27]. However, type I and type II cytokines, which are required to mediate the interaction between M1 and Th1, and between M2 and Th2 cells, respectively, are not included in order to keep the model structure relatively simple. The authors conclude that an M2/M1 macrophage ratio greater than one can explain the tumor size, but caution that experimental studies with longitudinal data on M2/M1 ratio biomarkers are too limited to confront these findings. Furthermore, data from human trials are also required to evaluate/validate the results.

The model proposed by Coletti et al. for prostate cancer successfully added compartmentalization into the prostate gland and lymphoid tissue to the structure using data from preclinical experiments, given the paucity of clinical studies [32]. The prostate gland compartment contains two types of prostate cancer cells, androgen-dependent (representing tumor cell sensitivity) and -independent (corresponding to tumor cell resistant) cells. Other players involved, connecting both compartments, are mature DCs, which in lymphoid tissues act as functional DCs (Df) or regulatory DCs (Dr) activating CTL or Treg, respectively. Finally, the pro-tumor immunosuppression exerted by MDSCs, an essential contributor to prostate cancer along with NK, androgens, and IL-2, have been included to explore different immunotherapies. Historically, prostate cancer has not responded well to immunotherapy possibly due to a strong immunosuppressive TME [33]. Thus, this model provides the framework to test in silico a variety of seven combinatorial immunotherapies: (1) androgen deprivation therapy, (2) cancer vaccines targeting mature dendritic cells, (3) anti-IL-2 for Treg inhibition, (4) anti-CD25 for Treg depletion, (5) administration of NK cells, (6) combination of anti-CTLA-4 with anti-PD-1 antibodies, and (7) cabozantinib (anti-MDSCs). A possible limitation of this model is the lack of effect of the suppressive molecules TGF-β and IL-10 secreted by Treg, which may exert a pro-tumoral effect. Given that, these biomarkers are easy to obtain from peripheral blood, suggesting their inclusion in future models. In addition, although beyond the scope of the current review focused on Treg function in the TME, cell mutation from androgen-dependent to androgen-independent prostate cancer cells may provide a novel aspect of QSP modeling in IO.

Finally, the most complex QSP model is reported by Ji et al. [34]. This model was developed for DTA-1.mIgG2a (DTA-1), a mAb agonist of GITR (glucocorticoid-induced tumor necrosis factor receptor-related protein), which is constitutively overexpressed on the surface of Treg. DTA-1, tested in two syngenic murine tumor models (CT26 and A20), has previously been shown to inhibit Treg and activate cytotoxic CD8^+^ T and NK cells. This is due to a dual activity: (1) inhibition of Treg by the binding of DTA-1 to GITR, and (2) a direct depletion of Treg by antibody-dependent cellular phagocytosis (ADCP). This depletion, mediated mainly by macrophages, downregulates IL-10 and allows for the activation of effector T cells influenced by IL-2 expression. To address this hypothesis, data consisting of serum concentrations of DTA-1, soluble GITR (sGITR), and anti-drug antibodies (ADAs) for pharmacokinetic (PK) assessment, as well as the major T cell subsets including intra-tumoral levels of Treg (distinguishing high and low GITR expression in Treg), effector CD8^+^ T cells (accounted as the sum of inactivated and activated effector T cells), and macrophages (FcgRIV) for pharmacodynamic (PD) assessment, were collected from different compartments and analyzed. DTA-1 PK was described by a two-compartment model with a non-linear elimination due to the presence of ADAs capable of binding to sGITR. DTA-1 plasma concentrations were directly connected with the tumor compartment for drug trafficking. Interestingly, the authors explain the tumor shrinkage as a result of the balance of the CD8^+^/Treg ratio, which first requires depletion of Treg capable of causing a reduction in the immunosuppressive effects of IL-10, then promoting the proliferation and differentiation of CD8^+^ T cells that eliminate cancer cells. Note that several complexities were addressed in this study, such as the different antitumor responses depending on the tumor model, and the optimal dose. To describe tumor elimination, the authors hypothesized that treatment responses were associated with the presence of Treg with high GITR expression, which can induce a greater ADCP effect. Although the model, under certain assumptions, successfully described the PK and PD of DTA-1 in the two preclinical tumor models, verification/validation using more complex experimental designs and the inclusion of other immune pathways will be necessary to understand/elucidate and quantify the mechanisms hypothesized here.

Remarkably, the dual activity assumed for DTA-1 is also shared by other mAbs against certain biomarkers expressed on Treg. This is the case for anti-CTLA-4, which can inhibit Treg activity and also induce Treg depletion via an ADCC mechanism, leading to an increase in antitumor efficacy. However, as activated effector T cells and DCs also express CTLA-4, this strategy could also cause a reduction in these immune cell populations, thereby hampering the activity of effector CD8^+^ T cells [35].

All the models reviewed have some characteristics that need to be highlighted. A shared limitation to models 1 to 4 is that they are all non-spatial tumor immune models, confined to the TME. Although the model proposed by Robertson-Tessi et al. was developed without spatial elements, it considers the number of tumor cells accessible to the immune system, linked with tumor size and vascularization. The spatial localization of immune cells may contribute to tumor growth and invasion, thus affecting the performance of the models, and therefore, the efficacy of immunotherapy, as mentioned by Ji et al. [34]. This highlights the need to compartmentalize the mathematical framework to take into account spatial heterogeneity. In addition, reciprocal modulations, which occur between different subpopulations such as Th1 and Th2, M1 and M2 or DCs at different stages of maturation, influencing the heterogeneity of the TME in terms of cytokine composition, is a limitation already pointed out by Den Breems et al. when developing their model. In this context, another important complexity to be considered is the plasticity and differentiation of lymphocytes into Th1, Th2, Treg, or Th17 profiles, highly adaptable to different TMEs, an event that requires serious efforts to be addressed quantitatively in order to be incorporated into QSP model structures [14,36].

Therefore, tumor types and TME heterogeneity may determine different responses to treatments as IO therapy does not achieve similar antitumor responses in all treated patients. Several internal and external factors are considered to explain the inter- and intra-tumor heterogeneity. New technologies, particularly the spatial transcriptomic applied to cancer research, offer a great opportunity to understand the localization of the different components of TMEs and tumor cells, which combined with the characterization of the diversity of gene expression and mutations, can explain the different phenotypes and thus the different therapeutic responses of tumors. In this context, the QSP approach is a valuable tool to integrate all information to explore diverse hypotheses and simulate potential combinations based on each immune patient profile. However, longitudinal preclinical and clinical data on subpopulations of pro- and antitumor cells are essential to fully assess the trade-off between Treg and CD8^+^ cells, which are responsible for IO efficacy. In this work, all models are based on CD25^+^ and Foxp3^+^ Treg, except for the model by Ji et al., which describes Treg subpopulations according to GITR expression levels. The relevance of the mentioned subpopulations was demonstrated by a transcriptomic analysis of 99 solid tumors from cancer patients enrolled in a clinical trial [37]. The authors analyzed the correlation between the expression of GITR and its endogenous ligand and the efficacy of several GITR agonists tested in preclinical and clinical trials. The heterogeneous expression of these proteins between and within cancer types evidenced the necessity to incorporate these data to achieve patient stratification to establish the most appropriate IO treatment. For example, tumor responders were those associated with high GITR and low–moderate GITR ligand expression, a phenotype found in approximately 30% of breast and lung cancer patients, respectively. However, this high GITR expression was independent of other immune modulators (PD-1, CTLA-4, OX40), suggesting that a more detailed analysis may be critical to provide rational combinations targeting different Treg biomarkers. The authors also concluded that other characteristics such as concomitant therapies and disease progression might be investigated in each patient as these may also influence the antitumor response. Overall, further studies are warranted to identify the most complete immune profile of patients to achieve efficacy.

In this context, QSP frameworks are useful tools in decoding cancer processes, providing an understanding of the disease, and evaluating the mechanism of action of molecular entities. The ultimate goal of these QSP models is to use virtual animal/patient populations to inform the design of clinical studies and to test in silico the efficacy of new treatments and combinatorial therapies [32]. The common conclusion of all the models described here, based mainly on preclinical data, is related to the extrapolation of the models to the clinical setting, highlighting the urgent need for the availability of clinical data to confront model predictions, as well as the inclusion of human parameters in QSP models. At this point, the QSP model reported by Ippolito et al. implements CD8/Treg and M1/M2 macrophage ratios to deal with tumor shrinkage using omics data to simulate virtual patients; meanwhile, more recently, Anbari et al. investigated potential biomarkers for uveal melanoma patients, identifying tumor and blood CD8^+^ T cell density, tumor CD8^+^/Treg ratio, and blood naïve CD4^+^ T cell density as key biomarkers [38,39]. However, these studies do not reproduce specific mechanisms around Treg-derived immune resistance effects influencing tumor growth.

**Table 1:** Summary of selected QSP models for describing the mechanisms derived from Treg in tumor growth (symbols: 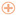 anti-tumor mechanisms; 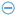 pro-tumor mechanisms).

	Immune Key Points	Treatment Evaluated	Model Structure
Cytokines controlling CD8^+^	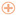 CD8^+^ mediates tumor killing and IFN-γ production.	IL-2 CD8^+^ cells	Kronik et al. [29] 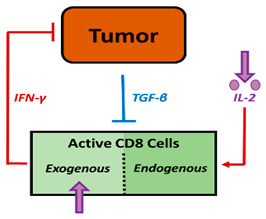
	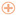 IFN-γ promotes MHC I expression on tumor cells for their recognition.		
	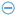 Inhibitory mechanisms are exclusively carried out by TGF-β produced by tumor cells.		
Pro-/Antitumor transition	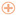 CD8^+^ accessible to tumor cells (effect of vascularization) exerts an antitumor effect.	DC therapy	Wilson et al. [30] 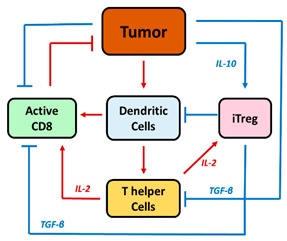
	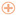 Key mechanism: Balance between antitumor effect derived from Dendritic cells (DC therapy) and pro-tumor effect exerted by iTreg.		
	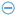 Inhibitory mechanisms: the presence of cytokines (IL-10 and TGF-β) and direct effect of iTreg.		
TGF-β as immunoresistance	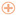 CD8^+^ and cancer vaccines are included separately, but both act synergistically as antitumor agents.	Cancer Vaccine Anti-TGF_β_	Robertson-Tessi et al. [31] 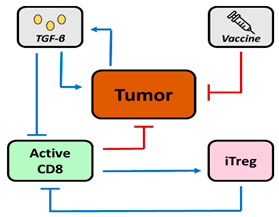
	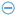 iTreg and TGF-β produced by tumors; both have inhibitory activity on CD8^+^ cells.		
M1/M2 macrophages control Tumor growth	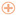 Antitumor activity exerted by M1 and type I cytokines (such as IFN-γ, IL-12, IL-2).	Simulations No treatment administration	Den Breems et al. [27] 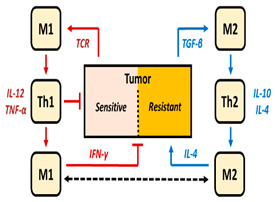
	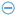 Immune resistance activity due to M2 and type II cytokines (such as TGF-β, IL-10, IL-4).		
Multiple immune cell trafficking across tumor and lymphoid tissues	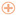 CD8^+^ and NK cells responsible for antitumor activity.	1: Androgen deprivation 2: Vaccine 3: Anti-IL-2 4: Anti-CD25 5: NK cells 6: anti-CTLA-4 + anti-PD-1 7: Cabozantinib (anti-MDSCs)	Coletti et al. [32] 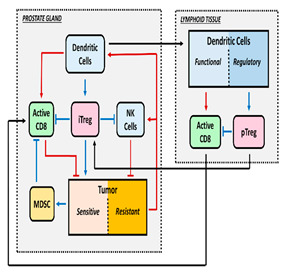
	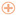 Dendritic cells stimulate CD8^+^ or Treg depending on their immune profile: DCfunctional/DCregulatory.		
	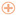 Immune cell trafficking across tumor and lymphoid tissues.		
	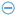 Pro-tumor activity is induced by MDSCs and Treg, both stimulated by tumor cells.		
GITR agonist inhibits Treg	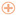 CD8^+^ elicited self-activation through IL-2 signaling after initial Treg depletion by therapy.	Anti-GITR	Ji et al. [34] 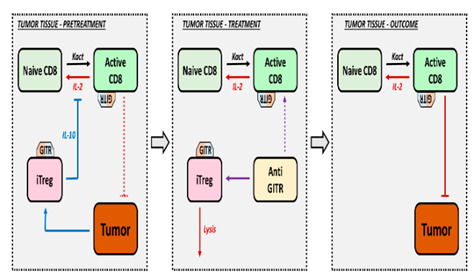
	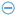 Treg with high GITR expression (Treghi) promote IL-10 secretion inhibiting CD8+.		

## 4. New Strategies for Treg Targeting

**Figure 3:**
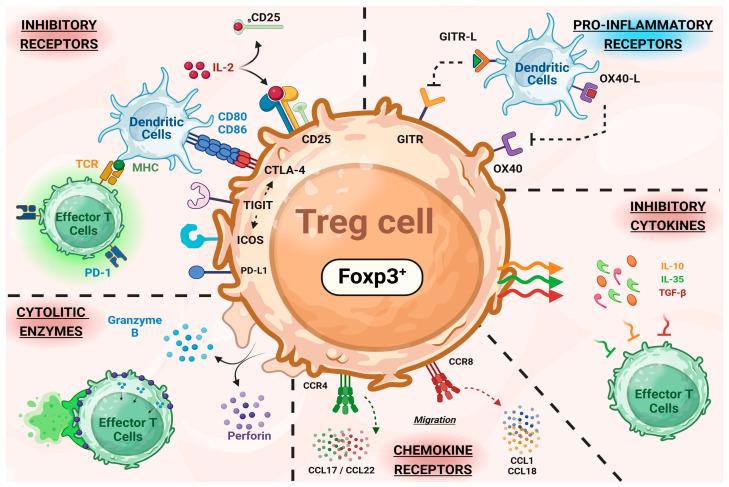
Schematic representation of the different biomarkers expressed in Treg and involved in the different mechanisms of action identified for this lymphocyte subset. Note that Foxp3 is expressed intracellularly and determines Treg activation.

Based on the QSP model proposed for Treg expressing GITR, other biomarkers such as CD25, CTLA-4, PD-1, ICOS, OX40, CCR4, and CCR8 (Figure 3) are useful targets that can also be exploited and integrated into similar QSP frameworks. Interestingly, mAbs that bind to OX40 (BAT6026, BGB-A445) or CCR8 (Nb-Fc1B) are being tested in preclinical models, as shown in Table 2. The expression of biomarkers in Treg therefore offers a wide range of possibilities for the development of therapies against immunosuppressive activity.

Notably, Treg targeting does not always guarantee the efficacy of immunotherapies. Therefore, a comprehensive integration of the mechanisms triggered by many of these therapeutic approaches to deal with Treg activity into a computational mathematical model can enrich the knowledge of immune activity and exquisitely improve the understanding of clinical benefits by predicting the most likely response according to different therapeutic combinations. Currently, some of these interesting advances in Treg modulation have reached clinical translation and are involved in several clinical trials (Table 3).

On the other hand, Foxp3, the transcription factor considered the master regulator of the Treg immunosuppressive phenotype, is an interesting biomarker that represents a challenge for the development of targeted therapies due to its intranuclear location. In this sense, intensive research has led to the spread of several peptides that reproduce parts of the Foxp3 amino acid sequences and compete for the binding partners and cofactors of Foxp3, or non-specific peptides with the ability to bind and inhibit Foxp3 activity [40]. Specifically, P60 is a linear peptide capable of selectively binding to Foxp3, preventing its nuclear translocation and inducing an antitumor response in preclinical tumor models. However, its very short systemic half-life requires a high daily dose administration, limiting its clinical translation.

To overcome this shortcoming, various modifications have been tested, such as the cycling of the linear structure or by the conjugation with the CD28 aptamer. Although these strategies provided a slight increase in the half-life of the peptide and an acceptable antitumor effect, other more efficient approaches are being sought. Serrano et al. [41] have reported a new nanosystem for P60 delivery. The authors have successfully encapsulated P60 in advanced nanoliposomes, which were decorated with monovalent variable fragments of anti-CD25 (Fab’-CD25) to selectively target Treg. This novel formulation was assayed in the MC38 mouse tumor model, and induced complete tumor remission in 40% of animals after monotherapy and in 100% after combination with anti-PD1. All mice developed immune memory. This result contrasts with the modest 10% of animals that showed tumor elimination after free P60 administration at a dose 50 times higher than that of the targeted liposome. Therefore, this nanosystem constitutes an interesting platform to exploit the transport and delivery of not only peptides but also other new molecules or treatments including different types of genes, i.e., small interfering-RNA (siRNA), which is capable of inhibiting specific gene expression [42]. Patisiran (Onpattro™), a lipid nanoparticle encapsulating siRNA, approved by the US Food and Drug Administration (FDA) in 2018 for the treatment of a rare metabolic disease, is a good example of nanosystems’ potential [43].

Selective therapies to modulate Treg activity, by depletion and/or inhibition, are therefore being actively investigated, with a particular focus on several biomarkers (Table 2 and Table 3). Interestingly, many clinical trials are using inhibitors against OX40, GITR, or LAG-3 with promising results. However, the availability of longitudinal data from these trials could help to propose and develop more complete QSP model structures that integrate pro- and antitumor mechanisms, allowing for the validation of model predictions, accelerating the approval of new therapies, and proposing the most rational combination depending on the individual immune profile. Computational models can also guide the design of clinical trials to improve the benefit of IO.

**Table 2:** Treg-targeted strategies developed and tested in preclinical studies.

Mechanism of Action	Target	Treatment	Concomitant Treatment	Tumor Cell Line	Immune Response	Ref.
Promotes Treg depletion	CD25 (IL-2Rα)	PC61 (mAb)	TLR9 agonist	Brain (E-L4)	↓ 45% Treg lymph nodes ** 30% Tumor regression ** OS 80% treated mice **	[44]
		PC61 (mAb)	Anti-CTLA-4	Melanoma (B16/BL6)	↓ 64% peripheral Treg in prophylaxis	[45]
	CTLA-4	4-E03 (mAb)	GM-CSF Anti PD-1	Cold tumors	↓ 82% inTreg tumor-infiltrating OS > 80% treated mice	[46]
	GITR	DTA-1 (mAb)	BMA-Ova (cancer vaccine)	Lung (3LL)	↑ CD8^+^ and NK in tumor * ↓ Tumor burden *	[47]
		DTA-1 (mAb)	--	Melanoma (B16)	OS 60% treated mice ** ↓ 50% Treg in tumor	[48]
		DTA-1 (mAb)	--	Urothelial (MB49)	↑ 82% CD8^+^ in tumor Total tumor regression 100% mice	[49]
	OX40	MEDI6383 (FP)	--	Melanoma (A375)	↓ Tumor burden * ↑ Proliferation of CD8^+^ in tumor	[50]
		BAT6026 (mAb)	Anti-PD-1	Colon (MC38)	↓ Tumor burden * ↑ 40% CD8^+^ in tumor ** ↓ 15% in Treg tumor-infiltrating	[51]
		BGB-A445 (mAb)	--	Colon (MC38)	↑ CD8^+^/Treg ratio in spleen ** ↓ Tumor burden *	[52]
	CCR8	Nb-Fc1B (NB)	Anti-PD-1	Colon (MC38) Lung (LLCOVA)	↓ Tumor burden ** OS (MC38) 15% treated mice OS (LLCOVA) 100% treated mice	[53]
		IgG2a (mAb)	Anti-PD-1	Solid Tumors	↓ Tumor burden ↓ 60% Treg in tumor *	[54]

OS, Overall Survival; mAb, monoclonal Antibody; FP, Fusion Protein; NB, Nanobody. (* *p* < 0.05; ** *p* < 0.001). ↓: Reduce, ↑: Increase.

**Table 3:** Treg-targeted strategies involved in clinical trials.

Mechanism of Action	Target	Treatment	Concomitant Treatment	Clinical Trial	Clinical Outcome	Ref.
Promotes Treg depletion	CD25 (IL-2Rα)	Daclizumab (mAb)	HLA-A2 (cancer vaccine)	FDA Approved	1st dose ↓ 70% Treg at weak 11	[55]
		Basiliximab (mAb)	ACT with CD8^+^ Cells	FDA Approved	1st dose ↓ 70% Treg-CD25^hi^ at day 7 *	[56]
		Denileukin difitox (FP)	--	FDA Approved	1st dose ↓ 25% Treg-CD25^hi^ at day 5	[57]
		LMB-2 (FP)	MART-1 (cancer vaccine)	NCT00080535	1st dose ↓ 70% Treg-CD25^hi^ for 7 days *	[58]
		RFT5-dgA (Immunotoxin)	--	NCT00314093 NCT00667017 NCT00586547	1st dose ↓ 100% Treg-CD25^hi^ for 7 days	[59]
	CCR4	Mogamulizumab (mAb)	Nivolumab	FDA Approved	Control Disease: 40% treated patients	[60]
Promotes Treg inactivation	CTLA-4	Tremelimumab (mAb)	--	FDA Approved	Control Disease: 51% treated patients OS increases in 2.8 months	[61]
		Ipilimumab (mAb)	Nivolumab Cisplatin	FDA Approved	60% Tumor Growth inhibition ** 15% treated patients increase OS	[62]
	PD1	Pembrolizumab (mAb)	Lenvatinib	FDA Approved	↓ Tumor burden ** 70% treated patients increase OS **	[63]
		Nivolumab (mAb)	Fluvestrant Letrozole	NCT01783938 NCT01176461	Control Disease: 40–55% treated patients	[64]
	LAG-3	Relatlimab (mAb)	Nivolumab	NCT03470922	40% treated patients increase OS	[65]
Promotes Treg inactivation	GITR	TRX518 (mAb)	Gembicatine Pembrolizumab Nivolumab	NCT01239134 NCT02628574 NCT03861403	Control disease: 30–50% treated patients OS increases in 2.6 months	[65]
		MK-1248 (mAb)	Pembrolizumab	NCT02553499	Control disease: 47% treated patients	[66]
		MEDI1873 (FP)	--	NCT02583165	↑ IFN-γ and Granzyme Stable disease: 42.5% treated patients	[67]
		GWN323 (mAb)	Spartalizumab	NCT02740270	Control disease: 34% treated patients	[68]
	OX40	Ivuxolimab (mAb)	Utomilumab	NCT02315066 NCT03971409 NCT03390296	Control disease: 34% treated patients Stable disease for 4–6 months	[69]
		GSK3174998 (mAb)	Pembrolizumab	NCT02528357	Control disease: 23% treated patients	[70]
		BMS-986178 (mAb)	Nivolumab Ipilimumab	NCT02737475 NCT03831295 NCT02737475	Control disease: 73% treated patients	[71]
		MEDI6469 (mAb)	--	NCT02559024 NCT02205333 NCT01862900	82% treated patients increase OS	[72]
		MOXR0916 (mAb)	--	NCT02410512 NCT02219724 NCT03029832	Control disease: 33% treated patients	[73]
		MEDI0562 (mAb)	Nivolumab Pembrolizumab	NCT03336606 NCT02705482 NCT02318394 NCT03267589	47% treated patients increase OS	[74]

OS, Overall Survival; mAb, Monoclonal Antibody; FP, Fusion Protein; ACT, Adoptive Cell Therapy; Control Disease encompasses patients who have achieved a complete response, a partial response, or a stable disease, all of which are defined according to the RECIST criteria (* *p* < 0.05; ** *p* < 0.001). ↓: Reduce, ↑: Increase.

## 5. Conclusions

Model-based quantitative pharmacology has demonstrated an important role in the development of new therapeutic molecules, providing information on doses and regimens and also exploring different mechanisms involved in tumor progression and elimination. QSP models applied to IO allow for the elucidation of specific immune mechanisms to better understand the biology of the disease, therapies, and individual immune profiles, with the aim of identifying different cancer types and patients to propose particular combinations to achieve an efficient antitumor response.

This review presents a list of diverse computational mathematical models that examine the role of regulatory T cells as a crucial immune resistance mechanism, contributing to the failure of immunotherapy. In all models, both Treg and cytotoxic CD8^+^ T cells are included, either explicitly or implicitly, in the quantitative immune frameworks proposed for evaluating tumor growth based on the immune therapies tested in preclinical models. While QSP models have demonstrated their capacity to explore intriguing hypotheses, longitudinal preclinical and clinical data on pro- and antitumor cell subpopulations are indispensable for identifying the most plausible mechanisms involved in IO. Furthermore, new strategies for targeting Treg based on specific biomarkers involved in their activation status are emerging as promising therapies to enhance the antitumor immune response, thereby generating considerable interest in many of them.

However, in order to individualize treatments and improve the efficacy of IO, QSP models face important challenges, such as the integration of data on tumor heterogeneity, the spatial localization of immune cells within the TME, the plasticity and activation status of immune cells, the identification of specific biomarkers, pro- and anti-inflammatory cytokine levels, and others. This will require more refined preclinical and clinical trial designs for longitudinal data collection and the application of new technologies.

## Data Availability

Data can be made available on request to corresponding authors.

## Supplementary Materials

The following supporting information can be downloaded at: https://www.mdpi.com/article/10.3390/pharmaceutics16111461/s1, Table S1: Classification of Treg cells based on the expression of biomarkers [75,76,77].

## Abbreviations

Adoptive Cell Therapy (ACT), antibody-dependent cellular cytotoxicity (ADCC), cytotoxic T lymphocytes (CTLs), Cytotoxic T-Lymphocyte-Associated Protein 4 (CTLA-4), Cluster of Differentiation 25 (CD25), dendritic cells (DC), Forkhead box P3 (Foxp3), Food and Drug Administration (FDA), Fusion Protein (FP), glucocorticoid-induced TNFR-related protein (GITR), Granulocyte-Macrophage Colony-Stimulating Factor (GM-CSF), Interleukin-2 (IL-2), Lymphocyte Activation Gene-3 (LAG-3), major histocompatibility complex I (MHC I), Monoclonal Antibody (mAb), Natural Killer cells (NK), Overall Survival (OS), Programmed Death-1 (PD-1), regulatory T cells (Treg), Response Evaluation Criteria in Solid Tumors (RECIST), small interfering RNA (siRNA), Tumor-Associated Macrophages (TAMs), and tumor microenvironment (TME).
