# Supplementary material for: Quantitative Approach to Explore Regulatory T Cell Activity in Immuno-Oncology
Source: Pharmaceutics. 2024 Nov 15;16(11):1461. doi: 10.3390/pharmaceutics16111461 (PMC11597491; doi:10.3390/pharmaceutics16111461)
Supplement: Supplementary file 1 [file pharmaceutics-16-01461-s001.zip › pharmaceutics-3264526-supplementary.pdf]

Supplementary Material

Table S1. Classification of Treg cells based on the expression of biomarkers [75–77].

Treg cell subset	Immune profile	Immune response (antitumor)	Mechanism of action
Non-Treg	$CD25^{low}CD45RA^{-}FOXP3^{low}$	Activator	Activated effector T cells expressing transiently CD25 and Foxp3.
Naive Treg	$CD25^{low}CD45RA^{+}FOXP3^{low}$	None	Naive without activation and/or differentiation.
iTreg	$CCR4^{+}CCR8^{+}FOXP3^{hi}$	Suppressor	Tumor-infiltrating Treg promoted by chemokine pathway activation.
	$CD4^{+}CD25^{+}CTLA\text{-}4^{+}GITR^{+}$		Treg highly immune-suppressive activity into TME.
pTreg	$Helios^{-}$ $Tigit^{-}$ *Treg*	Activator	Unstable peripheral Treg population whose phenotype may drift towards a $CD8^{+}$ profile with antitumor activity.
